# Contribution of *Podoviridae* and *Myoviridae* bacteriophages to the effectiveness of anti-staphylococcal therapeutic cocktails

**DOI:** 10.1038/s41598-020-75637-x

**Published:** 2020-10-29

**Authors:** Maria Kornienko, Nikita Kuptsov, Roman Gorodnichev, Dmitry Bespiatykh, Andrei Guliaev, Maria Letarova, Eugene Kulikov, Vladimir Veselovsky, Maya Malakhova, Andrey Letarov, Elena Ilina, Egor Shitikov

**Affiliations:** 1 Federal Research and Clinical Center of Physical-Chemical Medicine, Moscow, Russia; 2 Research Center of Biotechnology of the Russian Academy of Sciences, Winogradsky Institute of Microbiology, Moscow, Russia

**Keywords:** Antimicrobials, Bacteriophages, Clinical microbiology, Virology, Microbiology, Genetics, Genome

## Abstract

Bacteriophage therapy is considered one of the most promising therapeutic approaches against multi-drug resistant bacterial infections. Infections caused by *Staphylococcus aureus* are very efficiently controlled with therapeutic bacteriophage cocktails, containing a number of individual phages infecting a majority of known pathogenic *S. aureus* strains. We assessed the contribution of individual bacteriophages comprising a therapeutic bacteriophage cocktail against *S. aureus* in order to optimize its composition. Two lytic bacteriophages vB_SauM-515A1 (*Myoviridae*) and vB_SauP-436A (*Podoviridae*) were isolated from the commercial therapeutic cocktail produced by Microgen (Russia). Host ranges of the phages were established on the panel of 75 *S. aureus* strains. Phage vB_SauM-515A1 lysed 85.3% and vB_SauP-436A lysed 68.0% of the strains, however, vB_SauP-436A was active against four strains resistant to vB_SauM-515A1, as well as to the therapeutic cocktail per se. Suboptimal results of the therapeutic cocktail application were due to extremely low vB_SauP-436A1 content in this composition. Optimization of the phage titers led to an increase in overall cocktail efficiency. Thus, one of the effective ways to optimize the phage cocktails design was demonstrated and realized by using bacteriophages of different families and lytic spectra.

## Introduction

The wide spread of multidrug-resistant (MDR) bacterial pathogens is recognized by the World Health Organization (WHO) as a global threat to modern healthcare^1^. One promising alternative for the antibiotics to treat MDR infections is an approach that uses specific virulent bacteriophages as an antibacterial agent, known as phage therapy (PT). Virulent bacteriophages are natural predators of bacteria, highly specific, and independent of bacterial resistance to the antibacterial drugs^2^. Historically, phages as therapeutic agents have been used for almost a century mainly in the Eastern European countries in humans. Nowadays, a lot of reports from all over the world have been published about the successful use of bacteriophages in human health. Leading centers for the use of bacteriophages in therapy are located in Georgia, Poland, Belgium, and the USA. In turn, a growing network of phage biotech companies [MicroGen (Russia), Micro World (Russia), Eliava (Georgia), Pherecydes Pharma (France), Advanced Phage Therapeutics (USA), AmpliPhi Biosciences (USA)], as well as academic institutions, assist in the production process and/or supply of phages for use in therapy.

Therapeutic phage cocktails predominantly consist of a mixture of different bacteriophages directed against one or several bacterial species^3^. The most widely represented therapeutic cocktails are applied in case of infection caused by *Pseudomonas aeruginosa*, *Escherichia coli*, *Klebsiella pneumonia*, *Staphylococcus aureus* and other pathogens. Most promising results are obtained with virulent phages of *S. aureus*^3–5^. This pathogen causes different infectious processes (skin infection, toxin-related diseases, osteomyelitis, catheter-associated infection, and others) manifested by various symptoms ranging from relatively mild to life-threatening^6^. A significant fraction of *S. aureus* strains typed in routine clinical testing is resistant to beta-lactam antibiotics (oxacillin, methicillin) (the so-called methicillin-resistant *S. aureus* (MRSA)). MRSA incidence, reported by 85 (44%) of the WHO member states, exceeds 20% and reaches as high values as 80% in some of these countries^7^. For this reason, vancomycin became one of the first-line drugs to treat MRSA infections, but the clinical isolates of *S. aureus* with intermediate and complete resistance to vancomycin have emerged within the past two decades^8^. Alternatively, linezolid and tedizolid can be used, but they have major side effects including thrombocytopenia, neuropathy, and even optic neuropathy^9^.

Bacteriophages as an alternative to antibiotics in the treatment of staphylococcal infections were used for a long time, so significant data has been accumulated on the interaction of staphylophages with their host^10–12^. Safety in the use of virulent staphylococcal bacteriophages has been shown in numerous studies in animal experiments^13,14^. The application of bacteriophages to treat patients with MRSA infections has also been quite successful^15,16^. A multicenter, randomized controlled study in a population of patients with severe clinical conditions has been conducted with a phage cocktail produced according to good manufacturing practices^17^. The successful use of a good manufacturing practice for the preparation of virulent staphylococcal bacteriophages for intravenous administration was also reported^18^.

The compositions of several therapeutic phage cocktails active against *S. aureus*, as well as individual bacteriophages from various sources, have been characterized in numerous genomic and metagenomics studies^3,4,19^.

Most frequently, the main components of the cocktails are phages belonging to the *Myoviridae* family, sometimes viruses of the *Podoviridae* family are also present in these compositions^3,4,19^. The NCBI database contains information about 69 genomes of staphylococcal virulent bacteriophages (26 genomes of *Podoviridae* phages and 43 genomes of *Myoviridae* phages). *Staphylococcus aureus* phages feature broad host range and highly lytic capabilities, explaining their high efficiency in therapeutic cocktails^3^. However, the contribution of each bacteriophage in the composition of the therapeutic cocktail to the overall lytic activity of the cocktail is usually not evaluated. This can be related to an old practice of cocktail preparation when a number of bacterial strains were cultivated together and inoculated with a mix of phages, so the resulting cocktail composition was dictated by racing conditions between phage lines and bacterial hosts during infection.

In the present study, two *S. aureus* phages (podovirus and myovirus) isolated from the commercial *Staphylococcus* bacteriophage cocktail produced by Microgen (Russia) were characterized. The lytic activity of the commercial cocktail was compared with the host ranges of individual bacteriophages, the reasons for the differences in their efficiency were described. Finally, a reasonable approach was proposed to optimize the composition of commercial bacteriophage cocktails active against *S. aureus.*

## Results

### Isolation of the bacteriophages and their morphology

We determined the plaque forming activity of the commercial therapeutic cocktail produced by the Microgen Company (Russia), on the panel of 75 characterized *S. aureus* clinical isolates (Table S1). The lytic activity of the cocktail covered 85% of the test panel (Table 1).

**Table 1:** Host range analysis.

Phage or therapeutic cocktail	Sensitive strains (%)		
	*S. aureus*	*S. epidermidis*	*S. haemolyticus*
vB_SauM-515A1	64 (85.3%)	6 (13.3%)	0
vB_SauP-436A1	51 (68.0%)	0	0
vB_SauM-fRuSau02	63 (84.0%)	5 (11.1%)	0
The Staphylococcus bacteriophage cocktail (batch P332)	64 (85.3%)	6 (13.3%)	0
The mixture of vB_SauM-515A1 and vB_SauP-436A1	68 (90.6%)	6 (13.3%)	0

The bacteriophage vB_SauM-515A1was isolated from a plaque formed on the *S. aureus* strain SA515, which was sensitive to cocktail action. The bacteriophage vB_SauM-515A1 was purified by repeated single plaque isolation and its host range was determined on the same panel of the host strains. The lytic activity of this phage turned to be identical to the range of activity of the original commercial cocktail. However, when 11 *S. aureus* strains remained resistant to both the original cocktail and to the phage vB_SauM-515A1 isolated from it.

To search if some minor components that may be active against these 11 strains are present in the cocktail, we set up the enrichment cultures with the same commercial phage preparation with each of the 11 *S. aureus* strains. The phages active against four more strains were obtained. The ten plaques formed on each of these four strains were tested (using the toothpick transfer) on all the four hosts as well as on the strain SA515. All the plaques produced growth on each of the 4 strains, resistant to the cocktail, at the same time the cocktail-sensitive strain SA515 did not support the growth of any of these phages. The bacteriophages were purified from each of the four host strains by triple single plaque isolation. The subsequent analysis of the host range analysis of these phage isolates did not reveal any differences between them, so we considered these isolates identical. Therefore, we used for subsequent work one phage isolate obtained from the direct plating of the cocktail, namely the phage vB_SauM-515A1, and one phage isolate from the same cocktail using the cocktail-resistant SA436 strain; hereafter phage vB_SauP-436A1.

**Figure 1:**
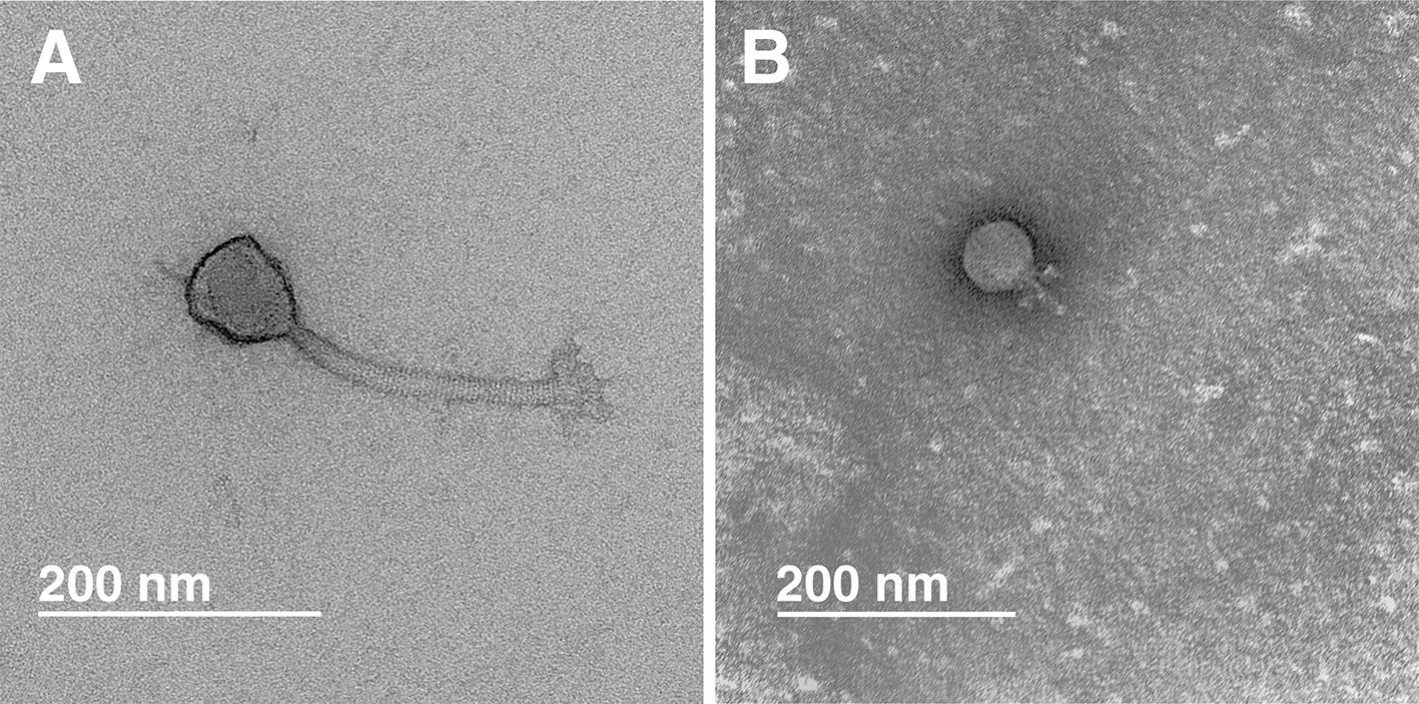
Transmission electron microscopy of vB_SauM-515A1 (**A**) and vB_SauP-436A1 (**B**).

The morphology of the phages vB_SauM-515A1 and vB_SauP-436A1 was determined by transmission electron microscopy. Phage vB_SauM-515A1 was a myovirus with an icosahedral head (82 ± 4.1 nm) and a contractile tail (199 ± 9.9 nm) (Fig. 1A). Electron microscopy of vB_SauP-436A1 phage showed that it is a podovirus with an icosahedral head (60 ± 3 nm) and a short, non-contractile tail (35 ± 1.8 nm) (Fig. 1B), a morphology resembling other staphylococcal *Podoviridae* viruses^20^.

### Genome analysis of vB_SauM-515A1 and vB_SauP-436A1

#### Genetic features of vB_SauM-515A1

The linear double-stranded DNA of phage vB_SauM-515A1 was 148,511 bp in length with two long terminal repeats (LTRs) of 7653 bp at both ends. At the extremities of the non-redundant part of the virion encapsidated genome, the inverted repeats of 12 bp sequence (5′-TAAGTACCTGGG-3′ and 5′-CCCAGGTACTTA-3′) were found, which is a characteristic feature of Twort-like viruses^21^. The analysis of predicted restriction endonuclease sites in the genome revealed that the sites for the enzymes of staphylococcal origin were mostly missing. Phage vB_SauM-515A1 genome completely lacked GATC sites for the Sau3AI enzyme and had only one Sau96I site (GGNCC) in its genome.

The complete genome of phage vB_SauM-515A1 contained 238 putative open reading frames (ORFs) and 4 tRNAs (tRNA-Met, tRNA-Trp, tRNA-Phe, and tRNA-Asp) (MN047438.1). The majority of genes (167 of 238 ORFs) are apparently transcribed from the positive strand and the remaining (located in the region from 7.8 to 40.8 kb) from the negative strand. Comparison of the predicted ORFs with available in public databases annotations did not reveal any genes for novel proteins, as well as genes of integrases, toxins, and virulence-associated factors. A group I introns were identified in genes encoding lysine, terminase, DNA polymerase-associated exonuclease, and RecA-like recombinase, which has been demonstrated for *Myoviridae* family phages previously^4,21^.

**Figure 2:**
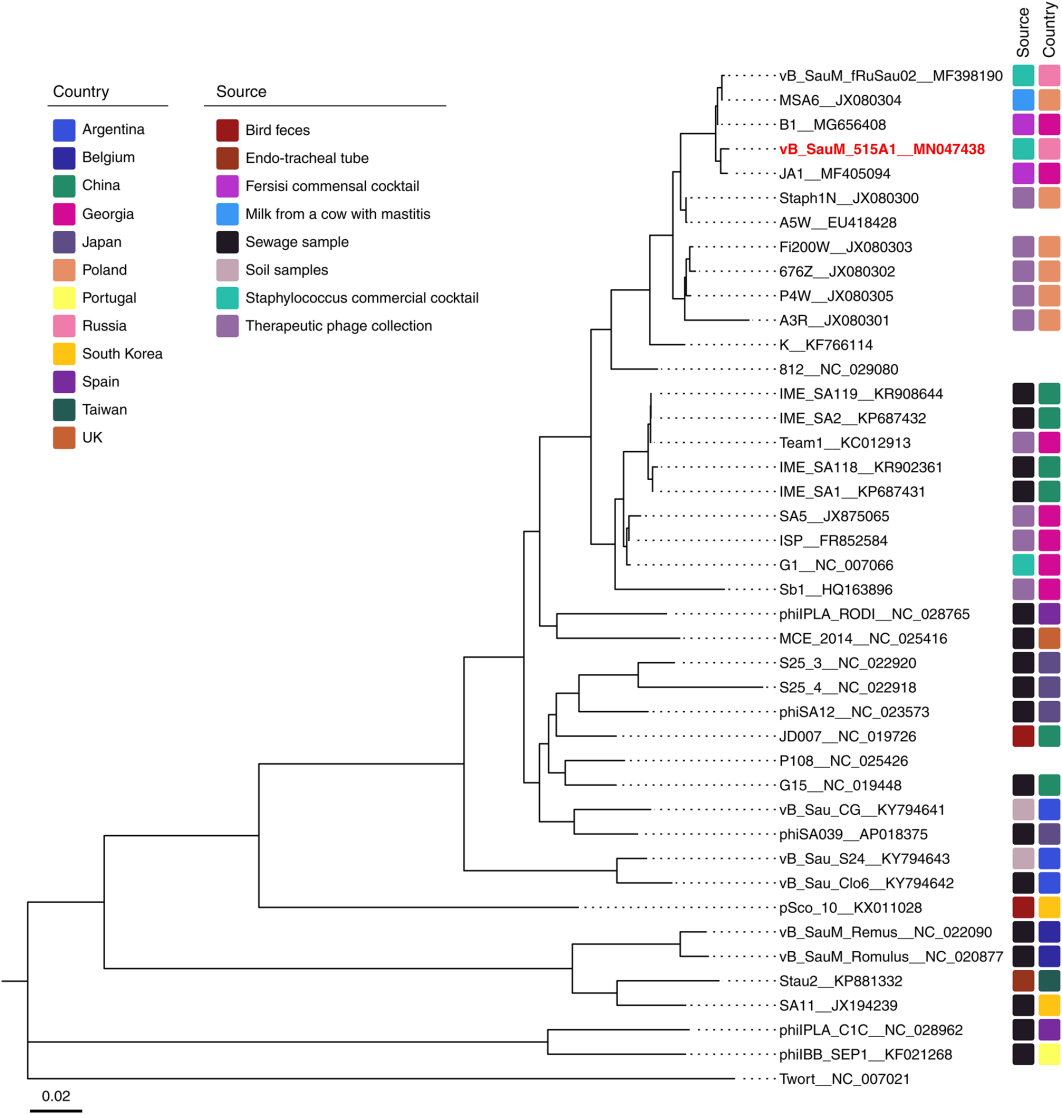
Phylogenetic tree of 42 staphylococcal phages, belonged to the *Myoviridae* family. The isolation source and country of each species are annotated with a colored rectangle, if no such information available rectangle is left blank.

The genome-based phylogenetic analysis of vB_SauM-515A1 and 41 phage genomes revealed clusterization with other Twort-like viruses. At the phylogenetic and genomic levels, our phage was closest to the vB_SauM_fRuSau (99.82%), MSA6 (99.78%), B1 (99.91%), and JA1 (99.89%) bacteriophages (Fig. 2). The genomic comparison of vB_SauM-515A1 with phages K and Twort showed identity rates of 99.49% and 70.98% respectively.

#### Genetic features of vB_SauP-436A1

The phage vB_SauP-436A1 genome was a 18,028 bp contiguous sequence of linear double-stranded DNA with an overall G+C content of 29.34%. Twenty-one ORFs were defined as potential phage genes, further categorized into four functional groups: structural (tail fibers protein, collar protein, capsid, and scaffold protein), lysis (*N*-acetylmuramoyl-l-alanine amidase, holin, amidase), DNA metabolism (single-stranded DNA-binding protein, DNA polymerase) and DNA packaging (MN150710.1). As in the case of the *Myoviridae* phage, all found genes had homologs in the NCBI database. No tRNAs were predicted in the genome.

**Figure 3:**
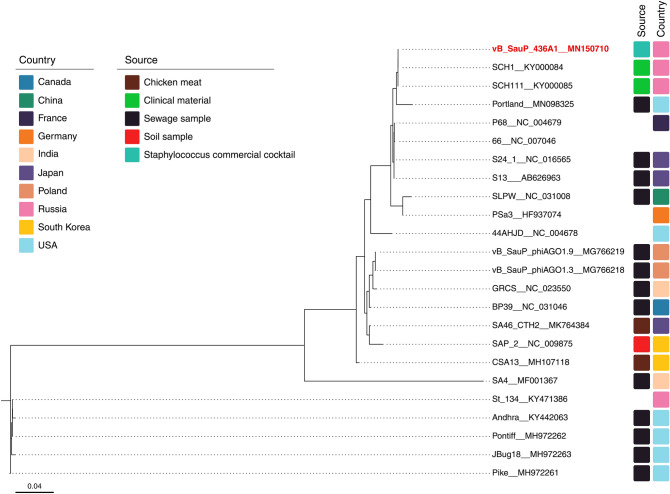
Phylogenetic tree of staphylococcus phages belonged to the *Podoviridae* family. The isolation source and country of each species are annotated with a colored rectangle, if no such information available rectangle is left blank.

Comparison with other *Podoviridae* phages available in the NCBI database showed that the vB_SauP-436A1 genome was closely related to genomes SCH1 (99.98%) and SCH111 (99.96%) (Fig. 3). The phages SCH1 and SCH111 were also isolated in Russia and had only two SNPs in comparison to the vB_SauP-436A1 phage genome. SCH111 additionally had three deletions in the genome, located in the polyA homopolymeric regions. The nearest phage Portland had more than 1000 SNPs and differences in the annotation of 11 ORFs relative to vB_SauP-436A1.

### Phages host range determination

A collection of 134 *Staphylococcus* strains, consisting of 75 *S. aureus* strains and 59 coagulase-negative *Staphylococcus* (CoNS) strains (45 *S. epidermidis* strains and 14 *S. haemolyticus* strains), was used to assess the host range (Table S1). *S. aureus* strains belonged to 26 different spa-types and t008 was the most frequent type (n = 21, 27.6%). The tested set included 36 MRSA and 40 MSSA strains. *S. epidermidis* and *S. haemolyticus* strains were characterized by MLST and belonged to 17 and 10 STs, respectively. Prevalent STs were ST59 for *S. epidermidis* (n = 14, 31.1%) and ST12 for *S. haemolyticus* (n = 4, 28.6%)*.*

The host ranges of vB_SauM-515A1 and vB_SauP-436A1 bacteriophages were compared with the lytic activity of the commercial therapeutic cocktail [the *Staphylococcus* bacteriophage cocktail (batch P332)] from which they were isolated. The host range of vB_SauM-fRuSau02 bacteriophage previously isolated from another Microgen therapeutic cocktail^4^ was also examined (Tables 1, S1). As expected, individual bacteriophages and phage cocktail showed a broad lytic activity against *S. aureus* strains (ranged from 68 to 85.3%), which was independent of drug resistance and spa-type. In turn, most CoNS strains were resistant to bacteriophages (Tables 1, S1).

It should be noted that phage vB_SauM-515A1 and *Staphylococcus* bacteriophage cocktail were equally effective and infected 64 of the 75 *S. aureus* strains. vB_SauM-fRuSau02 bacteriophage showed a similar breadth of lytic spectrum; only one additional *S. aureus* strain was resistant to vB_SauM-fRuSau02 phage. *Podoviridae* bacteriophage vB_SauP-436A1 had a narrower host range and was able to lyse 51 strains. Meanwhile, vB_SauP-436A1 phage was capable of forming plaques on the 4 strains of *S. aureus* resistant to *Myoviridae* phages and bacteriophage cocktail (Table S1).

The mixture of vB_SauM-515A1 and vB_SauP-436A1 containing equal PFU counts of these bacteriophages was prepared. The titers of phages in the mixture were 1.4 × 10^10^ PFU/ml. The lytic range of this mixture was broader than that of the original commercial phage cocktail from which both bacteriophages were isolated (Table 1). The mixture was able to infect all *Staphylococcus* spp. strains, which were sensitive to vB_SauM-515A1 and vB_SauP-436A1 separately [68 *S. aureus* strains (90.6%), 6 *S. epidermidis* strains (13.3%)] (Table 1). No plaque production was observed on *S. haemolyticus* strains.

### The analysis of the factors affecting the lytic activity of commercial cocktail

To evaluate the causes of reduced lytic activity of *Staphylococcus* bacteriophage cocktail, the following parameters have been analyzed: (1) titers of vB_SauM-515A1 and vB_SauP-436A1 in the cocktail; (2) biophysical stability of the bacteriophages; (3) infection parameters of bacteriophages; (4) efficiency of plating.

### Titers of vB_SauM-515A1 and vB_SauP-436A1 in the cocktail

The titer of vB_SauM-515A1 and vB_SauP-436A1 in the cocktail was evaluated on the host strains SA515 and SA436, respectively. As result, the titer of vB_SauM-515A1 bacteriophage was 10^4^-fold higher than that for vB_SauP-436A1 [8 × 10^6^ (PFU)/ml versus 2 × 10^2^ (PFU)/ml, respectively].

### Stability of the bacteriophages

To assess the biophysical stability of vB_SauM-515A1 and vB_SauP-436A1, the survival of bacteriophages at different temperatures and pH values was tested (Figure S1). Both bacteriophages were stable at 4 °C, the elevated temperature reduced their half-life with the greatest effect observed for the phage vB_SauP-436A1. In contrast, storage at − 20 °C resulted in more rapid inactivation of vB_SauM-515A1 phage particles (Figure S1). The incubation for 24 h at 4 °C at medium pH values ranging from 5 to 9 did not cause significant changes in the titer of the bacteriophages, while a pronounced alkaline (pH = 13) condition led to complete phage inactivation. A notable decrease of the infectious capacity of vB_SauM-515A1 and vB_SauP-436A1 was also observed after incubation at pH below 5 (Figure S1).

### Infection parameters of bacteriophages vB_SauM-515A1 and vB_SauP-436A1

**Figure 4:**
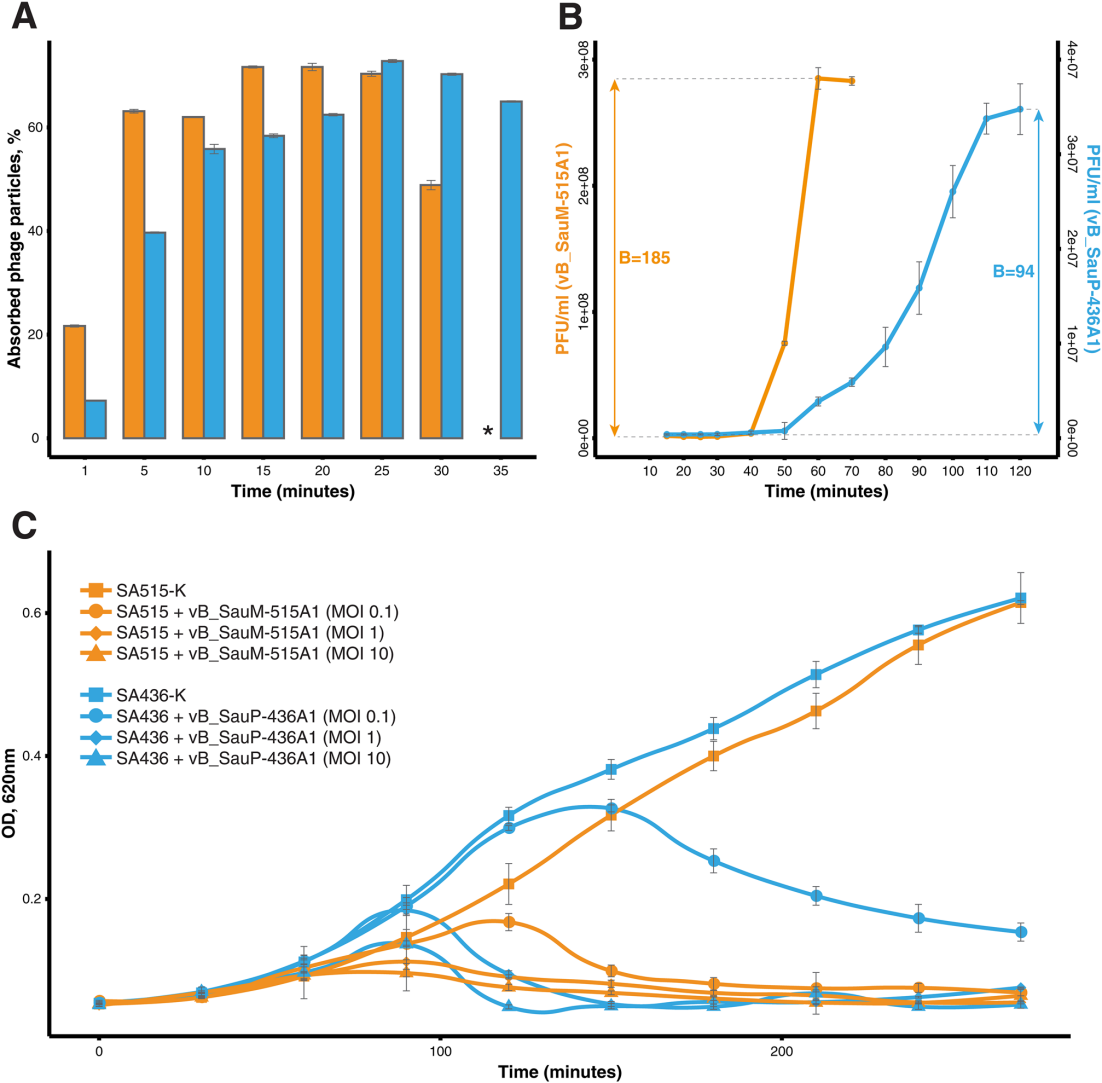
The infection parameters of vB_SauM-515A1 and vB_SauP-436A1. (**A**) The percentage of particles of vB_SauM-515A1 (orange bars) and vB_SauP-436A1 (blue bars) adsorbed on host strains surfaces. *The percentage of vB_SauM-515A1 particles on SA515 strain at 35 min exceeded 100%. (**B**) One-step growth curve analysis of vB_SauM-515A1 (orange bars) and vB_SauP-436A1 (blue bars) on an exponential culture of host strains. The corresponding burst size (**B**) values are given in numbers. (**C**) Growth curves of SA515 and SA436 strains infected with vB_SauM-515A1 and vB_SauP-436A1, respectively. The infection curves were obtained at different MOI values (0.1, 1, 10).

To examine the infection parameters of vB_SauM-515A1 and vB_SauP-436A1, the adsorption efficiency, latency period, burst size, and the infection efficiency were determined. The adsorption efficiency of the phages to host strains surfaces was evaluated at 0, 1, 5, 10, 15, 20, and 25 min (Fig. 4A). In accordance with the results, the phage vB_SauM-515A1 adsorbed on the bacterial cells faster (the maximum adsorption was observed at 15 min), then the vB_SauP-436A1 (the maximum adsorption was observed at 25 min), but the adsorption efficiency of these phages was comparable (vB_SauM-515A—71.7%, and vB_SauP-436A1—72.8%).

Results of a single-burst experiments shown in Fig. 4B revealed that vB_SauM-515A1 and vB_SauP-436A1 were characterized by different latency periods. Progeny phage particles were released after a 40 min post inoculation in the case of vB_SauM-515A1 and at 50 min in the case of vB_SauP-436A1. Also, the period of the release of the new phages by the SA436 culture infected by the phage vB_SauP-436A1 is much longer than for the vB_SauM-515A1 infection. This indicates that the latency period (the time from the infection to the cell lysis) may vary significantly from cell to cell, that is not the case for the SA515 culture infected by vB_SauM-515A1. We were not able to identify the molecular background for the observed differences in the cell lysis dynamics in these two phage-host systems.

The bactericidal effect of the phages vB_SauM-515A1 and vB_SauP-436A1 for SA515 and SA436 *S. aureus* cultures was evaluated based on the growth curves of host strains infected with the appropriate bacteriophages at different MOIs (Fig. 4C). Both bacteriophages were able to lyse the bacterial culture at MOIs 0.1–10. At the same MOI values, vB_SauM-515A1 caused lysis of the bacterial culture of the host strain quicker than vB_SauP-436A1, which is especially noticeable at low values of MOI. So bacterial culture SA515 was lysed by vB_SauM-515A1 at MOI 0.1 after 180 min (OD600 = 0.081), while bacterial culture SA436 infected by vB_SauP-436A1 had OD600 = 0.2533 at the same time. The optical densities of control cultures of SA515 and SA436 (without bacteriophages) at 180 min were 0.40 and 0.43, respectively.

The decreased burst size and the longer latent period of the phage vB_SauP-436A1 compared to vB_SauM-515A1 may explain the slower in vitro killing of the *S. aureus* culture by the former virus (Fig. 4C). Nevertheless, vB_SauP-436A1 causes the arrest of the culture growth and the decline of the optical density even at low MOI, which indicates that this phage may be applicable for the phage therapy.

### Efficiency of plating

The efficiency of plating (EOP) was evaluated for all *S. aureus* strains susceptible to both bacteriophages vB_SauM-515A1 and vB_SauP-436A1 according to the plaque assay data (n = 49) (Table S2). The EOP values of the vB_SauM-515A1 on these strains varied from 50 to 1000% (the average EOP value 224%) and significantly exceeded the EOP values of the vB_SauP-436A1 (the EOP value 1–433%; the average EOP value 133%). Lysis from without was revealed for 3 and 14 strains when interacting with bacteriophages vB_SauM-515A1 and vB_SauP-436A1, respectively.

## Discussion

The growing threat posed by multidrug-resistant bacteria has recently triggered interest in the development of phage therapy^22,23^. One of the advantages of the use of phages as antibacterial therapeutics is a fact that bacteriophages have high specificity. The therapeutic phage cocktail can affect only the microorganism that caused the infection and are expected to have little to no effect on normal microflora. On the other hand, the high specificity of bacteriophages often leads to their low efficiency. There are several ways to solve this problem, implemented at the stage of developing the therapeutic phage product, such as the use of multi-component phage mixtures and selection of circulating clinical strains of the target bacterium^22^.

Today the most common therapeutic phage products are the phage cocktails, which consist of a set combination of lytic phages to address the diversity within a single bacterial pathogen or multiple bacterial pathogens^22^. In this study, the contribution of individual bacteriophages that were part of commercial therapeutic cocktail active against *S. aureus* was assessed and the variant of optimizing the composition of the cocktails in order to increase its efficiency is described. According to the manufacturer, the bacteriophage cocktail under study can be used in the treatment and prevention of purulent infections of the skin, mucous membranes, visceral organs caused by staphylococcal bacteria, as well as for the treatment of dysbacteriosis.

Most staphylophages detected in commercial preparations belong to the *Myoviridae* family, namely to the *Kayvirus*^4,24^. The *Myoviridae* phages are associated with the lytic life cycle and characterized by the high efficiency of the bacterial lysis. They are most common in the environment and are isolated from various sources (Fig. 2). The presence of *Podoviridae* bacteriophages in the commercial therapeutic cocktails has been also shown in several studies^3^. The *S. aureus Podoviridae* bacteriophages are virulent phages, but they are less commonly occurring in cocktails than *Myoviridae* staphylophages. In this work, bacteriophages belonging to both families were isolated from the therapeutic cocktail produced by Microgen company. Isolated bacteriophages vB_SauM-515A1 (*Myoviridae* phage) and vB_SauP-436A (*Podoviridae* phage) were similar to the previously described bacteriophages of the corresponding families isolated from commercial cocktails. Based on the analysis of genome data, the most closely related to vB_SauM-515A1 was the vB_SauM_fRuSau bacteriophage, also isolated from the commercial therapeutic cocktail produced by Microgen company (the Staphylococcus bacteriophage cocktail; series: H52, 0813, PN001973/01). The SCH1 and SCH111 phages, which were also reported as a part of the therapeutic cocktail^25^, were the closest phages to vB_SauP-436A by the homology of nucleotide sequences.

The most important characteristic of a therapeutic phage cocktail is its lytic activity range that is determined by the host ranges of the individual bacteriophages present in the cocktail in considerable concentrations. *S. aureus* bacteriophages of both the *Podoviridae* family and the *Myoviridae* family are characterized by broad host ranges^11^, which is consistent with the data obtained in this study. But it was found that vB_SauP-436A1 had a narrower host range than vB_SauM-515A1. This fact is associated with differences in the mechanisms of adsorption of *S. aureus* bacteriophages belonging to the *Myoviridae* and *Podoviridae* families. *Podoviridae* phages use the β-*N*-acetylglucosamine residue in the *S. aureus* wall teichoic acid as a receptor^26,27^. Meanwhile, *M*y*oviridae* phages recognize the backbone of the wall teichoic acid^28^, and could infect a broader range of *S. aureus* strains. Moreover, vB_SauM-515A1, like one of *Myoviridae* phage, can even infect CoNS, in contrast to *Podoviridae* staphylophages^11^.

Despite the narrower host range vB_SauP-436A1 could lyse vB_SauM-515A1 insensitive *S. aureus* strains (n = 4, 5.3%). It should be noted that these strains were also resistant to the action of the vB_SauM_fRuSau bacteriophage belonging to the *Myoviridae* family. Similar data have been obtained in other studies, describing cases of lysis of a *Kayvirus-*resistant *S. aureus* strains with *P68 Podovirus*^3,29^*.*

Moreover, it was expected that the Microgen therapeutic cocktail should lyse *Staphylococcus* strains sensitive to each of the isolated bacteriophages, but was active only against the vB_SauM-515A1 sensitive strains. Although the Microgen therapeutic cocktail had a broad range of activity (85.3% of the strains tested), an extension of the activity over another 5.3% may turn crucial for the patients suffering from an infection caused by a multi-drug resistant strain belonging to this fraction. Two of *Myoviridae* insensitive strains were resistant to oxacillin and belonged to the prevalent spa-types characterizing to hospital strains (Table S1).

It was found that the reduced lytic activity of the Microgen therapeutic cocktail was associated with an extremely low titer of vB_SauP-436A1. The reduced concentration of this bacteriophage in the cocktail may be due to its instability or loss of titer in the process of the combined propagation of two bacteriophages (vB_SauM-515A1 and vB_SauP-436A1) in the manufacture of a therapeutic product. We suggest, that more rapid adsorption and a shorter latent period of the vB_SauM-515A1 phage compared to vB_SauP-436A1 (Fig. 4) could lead the former virus to outcompete the latter upon co-cultivation in the liquid medium.

A similar assumption about the presence of a “winner” phage in cocktails was made by the authors of a metagenomic study^3^. This highlights the need for separate cultivation of the bacteriophages to be included into the therapeutical preparation prior to their mixing in appropriate proportions—the approach advocated since the very early age of phage therapy^30^.

## Conclusions

In summary, we showed one of the effective ways to optimize the design of fixed phage cocktails. The data obtained clearly demonstrate the necessity of using bacteriophages of different families with different host ranges to produce an efficient cocktail. This synergy has been demonstrated in staphylophages belonging to the *Myoviridae* and *Podoviridae* families. The approach to developing optimized fixed phage cocktails does not preclude the use of alternative phage therapeutic formation variants, for instance, genetic engineering of phages for therapeutic purposes. And the B_SauM-515A1 and vB_SauP-436A1 bacteriophages included in fixed phage cocktails may become the basis for future genetically engineered bacteriophages.

## Materials and methods

### Bacterial strains

*Staphylococcus* isolates (n = 134) were collected from 24 hospitals in Russia. Isolates were recovered from several clinical sources, including the respiratory tract (sputum, pharynx swabs, and bronchial alveolar lavage fluid), skin and soft tissue (cutaneous abscess and wound secretion), cerebrospinal fluid, blood, and urine (Table S1). All the isolates received from clinical facilities were anonymized by the providers. The species identification was performed by MALDI-TOF mass spectrometry as described earlier^31^. In total, 75 *S. aureus* strains, 45 strains of *Staphylococcus epidermidis*, and 14 *Staphylococcus haemolyticus* strains were collected. The antibiotic susceptibility was determined according to Clinical and Laboratory Standards Institute (CLSI) recommendations using strain ATCC 43300 as a control. The following ten drugs were tested: oxacillin, vancomycin, chloramphenicol, ciprofloxacin, clindamycin, erythromycin, gentamicin, levofloxacin, linezolid, and tetracycline. All strains were grown in Luria Bertani (LB) broth or on LB agar plates at 37 °C.

### Typing of Staphylococcus strains

Bacterial DNA was isolated with a QIAamp DNA minikit (Qiagen, The Netherlands) according to the manufacturer's protocol. In *S. aureus*, the polymorphic X region of the staphylococcal protein A (*spa*) gene was amplified and sequenced according to Koreen et al.^32^. The spa-type was assigned by submitting the data to the *S. aureus* spa-type database (https://spaserver.ridom.de). Multi-locus sequence typing (MLST) of *S. aureus* strains was performed according to https://pubmlst.org/saureus. *S. epidermidis* and *S. haemolyticus* strains were genotypically characterized by MLST according to https://sepidermidis.mlst.net and Kornienko et al.^33^.

### Bacteriophage isolation and purification

Bacteriophages vB_SauM-515A1 and vB_SauP-436A1 were isolated from the commercial *Staphylococcus* bacteriophage cocktail (batch P332) produced by Microgen (Russia) using enrichment cultures^34^. *S. aureus* strains SA515 and SA436 of the aforementioned bacterial collection were taken as host strains of isolated bacteriophages (Table S1). Briefly, the bacteriophage cocktail was incubated with *S. aureus* host strain in LB broth overnight at 37 °C. The culture was centrifuged at 12,000*g* for 10 min, and the supernatant was collected and filtered with a 0.22 µm membrane (Merck Millipore; USA) to remove coarse bacterial debris. Then the supernatant was serially diluted in LB broth. Aliquots (100 µl) of these diluted phage suspensions, together with 100 µl of *S. aureus* culture, were mixed with 5 ml of soft top agar and poured on top of the solidified LB agar plates. The plates were incubated overnight at 37 °C to form plaques. Phage purification was repeated at least three times, and the final purified phages were then collected and stored at 4 °C. In addition, bacteriophage vB_SauM-fRuSau02 was used for comparison with isolated bacteriophages.

Phage titer was estimated by ten-fold dilution of the phage lysate in LB broth, 5 µl of each dilution was spotted on a plate containing 100 µl [10^6^ colony-forming unit (CFU)] of an overnight culture of host strain in 5 ml 0.4% (w/v) LB agar. Plates were incubated at 37 °C for 24 h with lysis plaques counted afterward. Phage titer was denoted in plaque forming units (PFU)/ml.

The crude lysates of isolated bacteriophages were purified on sucrose step gradient by ultracentrifugation. All steps were carried at + 20 °C. The lysates were cleared of debris by high-speed centrifugation [20,000*g*, Beckman JA-20 rotor (Beckman Coulter, USA)], supernatants containing free phage were sedimented at 75,000*g* [1 h, Beckman Type 45Ti rotor (Beckman Coulter, USA)]. Phage-containing precipitates were resuspended in 5% (w/v) buffered sucrose containing 50 mM Tris–HCl (pH 7.5) and 150 mM NaCl. The resulting phage suspension was layered on step gradient of buffered sucrose (20–30–40–50–60%) in Beckman SW55 5 ml centrifuge tubes. The gradients were centrifuged 30 min at 50,000*g*, and free phage particles formed a visible opalescent band between 40 and 50% steps. The overlaying steps containing debris and phage shadows were removed, and phage-containing bands were collected and dialyzed overnight at + 4 °C against Tris-buffered (10 mM, pH 7.5) physiological saline.

### Transmission electron microscopy

Purified phage preparations (vB_SauM-515A1 and vB_SauP-436A1) were analyzed by transmission electron microscopy using a JEOL JSM 100 CXII electron microscope (JOEL, Japan) at an acceleration voltage of 100 kV with a Gatan Erlangshem CCD camera (Gatan, Inc). Carbon-coated grids with collodion supporting film were negatively stained with 1% uranyl acetate in methanol.

### Bacteriophage DNA isolation and genome sequencing

Lysis buffer (final concentration: 0.1% sodium dodecyl sulfate, 20 mM EDTA, and 50 μg/ml proteinase K) was added to purified samples and incubated at 56 °C for 1 h. DNA was isolated by phenol/chloroform extraction method as described previously^35^. Whole genome sequencing of bacteriophages (vB_SauM-515A1 and vB_SauP-436A1A1) was performed with a high throughput Illumina HiSeq system sequencing. The phages genomes were completed by Sanger sequencing and deposited in the GenBank database (MN047438.1 and MN150710.1 for vB_SauM-515A1 and vB_SauP-436A1, respectively).

### In silico* analysis of phage genomes*

De novo assembly was performed using SPAdes (v.3.11.0)^36^. The phage genomes were autoannotated using Rapid Annotation Using Subsystem Technology (RAST)^37^, and on the basis of homology with previously described phages. The function of some ORFs was predicted by BLASTP (https://blast.ncbi.nlm.nih.gov/Blast.cgi) and HHpred (https://toolkit.tuebingen.mpg.de/#/tools/hhpred). Transfer RNA was found using ARAGORN^38^.

The genome-based phylogenetic analysis of genomes was performed using the VICTOR online tool^39^, a tree constructed with distance formula d_0_ was used for further analysis. Trees and plots were generated with ggtree (v.2.2.4) and ggplot2 (v.3.3.2) packages for R (v.4.0.2)^40–42^. Average nucleotide identity (ANI) was calculated with the pyani (v.0.2.10) tool [https://github.com/widdowquinn/pyani].

### Phage host range determination and the lytic activity of the commercial therapeutic phage cocktails

Host range determination of individual bacteriophages and the ability of therapeutic phage cocktails to produce plaques were performed by the plaque assay as previously described with some modifications^4,5,24^. Five microliters of phage lysate at a titer of 10^6^ PFU/ml or five microliters of the commercial therapeutic bacteriophage cocktail was spotted on indicator plates, which contained 100 µl of late log phase cultures of investigated strains (10^6^ CFU) and 5 ml of top LB agar (0.6% w/v). Plates were incubated overnight at 37 °C. The lytic activity was visually assessed by the appearance of the clear lysis zone at the points of application of the bacteriophages.

### Bacteriophage stability

The stability of the bacteriophages was examined as previously described^43^. In brief, the bacteriophage stocks were incubated at different temperature (− 20 °C, 4 °C, 37 °C, 42 °C, and 50 °C) for 24 h. After incubation, the samples were titrated and compared with control (4 °C).

The pH stability of phages was tested by diluting phage particles to a final concentration of 4 × 10^9^ PFU/ml (vB_SauM-515A1) and 4 × 10^8^ PFU/ml (vB_SauP-436A1) at pH ranged from pH = 3 to pH = 13. Phage suspensions were incubated for 24 h at 4 °C, then titrated and compared with control (pH = 8). Assays for the determination of the stability to temperature and pH were made in triplicate.

### Adsorption assay

The adsorption ability between phages and the host strains was evaluated as follows: bacterial cells and phages were mixed at MOI of 0.001 in a total volume of 1 ml. The mixture was incubated at 37 °C with shaking at 120 rpm. An aliquot of 10 μl was taken periodically at 0, 1, 5, 10, 15, 20, 25, 30, 35 min, and immediately diluted into 1 ml SM buffer. Then, samples were centrifuged at 10,000*g* for 2 min at 4 °C. The phage titer of the supernatants was evaluated by counting PFU from a full-size Petri dish when applying 100 µl of the bacteriophage suspension. The phage titer at time zero was determined as 100% of relative phage titer.

### The one-step growth curve and determination of burst-size

The one-step growth curve of phages on the host strains was performed in triplicates as described earlier^44,45^. The host strains were incubated until the early exponential growth phase (OD600 = 0.12). An aliquot of 990 μl of the cell cultures was mixed with 10 μl of the phage lysate, in order to achieve an MOI value of 0.001. The mixture was incubated for 7 min at 37 °C, and centrifuged at 10,000*g* for 4 min. The pellet was resuspended in 50 ml LB to remove any non-adsorbed phages and incubated at 37 °C, with shaking at 220 rpm. The aliquots of 10 μl were taken periodically at 15, 20, 25, 30, 40, 50, 60, and 70 min from the beginning of the infection. Samples were plated after treatment with 1% (vol/vol) chloroform. Subsequently, phage titers of the aliquots were evaluated by counting PFU from a full-size Petri dish when applying 10 µl of the bacteriophage suspension. The latency period was defined as the time between infection (including the 15 min of pretreatment) and the shortest incubation time allowing the production of phages. The burst size was defined as the number of phages released from each infected cell and calculated as a ratio of the final count of liberated phage particles to the initial count of infected bacterial cells during the latent period.

### Infection growth curve

The phage particles and bacterial cells were mixed to achieve different values of MOI (0.1, 1, 10) in 30 ml of LB. As a control, bacterial cells (OD 0.067) without the addition of phage particles were used. The mixture was incubated at 37 °C with shaking at 200 rpm. The optical density at 600 nm was measured every 30 min on Multiskan FC Microplate Photometer (Thermo Scientific, USA). Three independent experiments were carried out.

### Determination of efficiency of plating

In order to avoid false positive results associated with the effect of lysis from without and/or poor infectivity of the phage that precludes efficient virus multiplication and the plaque formation on some host strains, we determined the efficiency of plating of the cocktail and of the bacteriophage isolates as it was described earlier^46^. Briefly, the 5 μl drops of the ten-fold serial dilutions of phage lysates were applied onto the double-layer plate inoculated with 100 μl of the corresponding host strain culture (10^6^ CFU/ml). The phage determined on the isolation host strain was taken as 100%. Each assay was performed in triplicates. It was considered that the effect of lysis from without was observed if the effect of lysis was observed, but there were no single plaques in any of the dilutions.

## Supplementary information

Supplementary Information.
