# Supplementary material for: Contribution of Podoviridae and Myoviridae bacteriophages to the effectiveness of anti-staphylococcal therapeutic cocktails
Source: Sci Rep. 2020 Oct 29;10:18612. doi: 10.1038/s41598-020-75637-x (PMC7596081; doi:10.1038/s41598-020-75637-x)
Supplement: Supplementary file 1 — Supplementary Information. [file 41598_2020_75637_MOESM1_ESM.pdf]

SUPPLEMENTARY MATERIAL

Contribution of *Podoviridae* and *Myoviridae* bacteriophages to the effectiveness of anti-staphylococcal therapeutic cocktails

M. Kornienko[1], N. Kuptsov[1], R. Gorodnichev[1], D. Bespiatykh[1], A. Guliaev[1], M. Letarova[2], E. Kulikov[2], V. Veselovsky[1], M. Malakhova[1], A. Letarov[2], E. Ilina[1], E. Shitikov[1]

[1] Federal Research and Clinical Center of Physical-Chemical Medicine, Moscow, Russia

[2] Research Center of Biotechnology of the Russian Academy of Sciences, Winogradsky Institute of Microbiology, Moscow, Russia

Table S1 **2**

Table S2 **8**

Figure S1 **10**

Table S1. Characterization of *Staphylococcus spp* strains

Strain	Species	City	Origin	Drugs susceptibility										MLST	Spa type	The plaque assay results				
				Oxacillin	Chloramphenicol	Ciprofloxacin	Clindamycin	Erythromycin	Gentamicin	Levofloxacin	Linezolid	Tetracycline	Vancomycin			vB_SauM-515A1	vB_SauP-436A1	Staphylococcus bacteriophage cocktail	mixture of vB_SauM-515A1 and vB_SauP-436A1	vB_SauM_fRuSau
SA0090V	*S. aureus*	Seversk	conjunctiva	R	S	S	N/D	S	S	S	N/D	S	N/D		t759	-	-	-	-	-
SA0172REV	*S. aureus*	Moscow	nervous system injury	S	S	S	S	S	S	S	S	S	S		t435	-	+	-	+	-
SA0184REV	*S. aureus*	Moscow	endotracheal tube	S	S	S	S	S	S	S	S	S	S		unique 08-16-02-16-13-13-17-13-13	-	+	-	+	-
SA0210V	*S. aureus*	Samara	eyelid infection	S	S	S	N/D	S	S	S	N/D	S	N/D		unique 08-34-24-25-25	-	-	-	-	-
SA0412REV	*S. aureus*	Lipetsk	skin infection	R	R	S	R	R	R	S	S	I	S		t723	-	+	-	+	-
SA0606REV	*S. aureus*	Oryol	skin infection	S	S	S	S	S	S	S	S	S	S		t3625	-	-	-	-	-
SA0722REV	*S. aureus*	Tomsk	blood	S	S	S	S	S	S	S	S	S	S		t127	-	-	-	-	-
SA1550REV	*S. aureus*	Rostov-on-Don	abdominal space infection	S	S	S	S	S	S	S	S	S	S		t267	-	-	-	-	-
SA2013REV	*S. aureus*	Voronezh	skin infection	S	S	S	S	S	S	S	S	S	S		t3625	-	-	-	-	-
SA2060REV	*S. aureus*	Tolyatti	blood	S	S	S	S	S	S	S	S	S	S		t2361	-	-	-	-	-
SA0013BAS	*S. aureus*	Tumen	blood	R	R	S	R	R	R	S	S	R	S		t008	+	-	+	+	+
SA0037REV	*S. aureus*	Smolensk	skin infection	S	R	S	S	S	S	S	S	S	S		t435	+	+	+	+	+
SA0038REV	*S. aureus*	Smolensk	abdominal space infection	R	R	I	R	R	R	R	S	R	S		t190	+	+	+	+	+
SA0054V	*S. aureus*	Irkutsk	conjunctiva	S	S	S	N/D	S	S	S	N/D	S	N/D		t081	+	+	+	+	+
SA0064BAS	*S. aureus*	Saint Petersburg	blood	R	R	R	S	S	S	R	S	R	S		t008	+	+	+	+	+

SA0088ST	*S. aureus*	Krasnodar	skin wound	R	S	S	S	S	S	S	S	S	S		t308	+	+	+	+	+
SA0088V	*S. aureus*	Seversk	conjunctiva	S	S	S	N/D	S	S	S	N/D	S	N/D		t9374	+	+	+	+	+
SA0103BAS	*S. aureus*	Krasnodar	blood	S	S	S	S	S	S	S	S	S	S		t127	+	+	+	+	+
SA0117V	*S. aureus*	Vologda	eyelid infection	S	R	S	N/D	S	S	S	N/D	S	N/D		t435	+	+	+	+	+
SA0120V	*S. aureus*	Vologda	conjunctiva	S	S	S	N/D	S	S	S	N/D	S	N/D		t1407	+	+	+	+	+
SA0156V	*S. aureus*	Smolensk	conjunctiva	S	S	S	N/D	S	S	S	N/D	S	N/D		t267	+	+	+	+	+
SA0158V	*S. aureus*	Smolensk	conjunctiva	S	S	S	N/D	S	S	S	N/D	S	N/D		t267	+	+	+	+	+
SA0177REV	*S. aureus*	Moscow	endotracheal tube	R	R	I	R	R	R	R	S	R	S		t030	+	+	+	+	+
SA0187ST	*S. aureus*	Krasnodar	bones and joints infection	R	S	S	S	S	S	S	S	S	S		t021	+	-	+	+	+
SA0191REV	*S. aureus*	Moscow	spinal fluid	R	R	I	R	R	R	R	S	R	S		t030	+	+	+	+	-
SA0220V	*S. aureus*	Smolensk	conjunctiva	R	S	S	N/D	S	S	S	N/D	S	N/D		t267	+	+	+	+	+
SA0293REV	*S. aureus*	Voronezh	blood	S	S	S	S	S	S	S	S	S	S		t385	+	+	+	+	+
SA0326REV	*S. aureus*	Tomsk	lungs	R	R	R	R	R	R	R	S	S	S		t008	+	-	+	+	+
SA0346ST	*S. aureus*	Moscow	abscessus	R	S	S	R	R	R	S	S	R	S		t024	+	+	+	+	+
SA0372ST	*S. aureus*	Moscow	abscessus	R	R	S	R	R	R	S	S	R	S		t308	+	+	+	+	+
SA0385REV	*S. aureus*	Yekaterinburg	bronchoalveolar lavage	S	S	S	S	S	S	S	S	S	S		t015	+	+	+	+	+
SA0395ST	*S. aureus*	Moscow	skin infection	R	S	S	S	S	S	S	S	S	S		t1023	+	+	+	+	+
SA0402REV	*S. aureus*	Lipetsk	bones and joints infection	R	R	R	R	R	R	R	S	R	S		t233	+	+	+	+	+
SA0413REV	*S. aureus*	Lipetsk	skin infection	S	R	I	R	R	R	R	S	R	S		t008	+	+	+	+	+
SA0414REV	*S. aureus*	Lipetsk	skin infection	R	R	R	R	R	R	R	S	R	S		t008	+	-	+	+	+
SA0422ST	*S. aureus*	Nizhny Novgorod	skin wound	R	R	S	S	S	R	S	S	R	S		t008	+	+	+	+	+
SA436	*S. aureus*	Nizhny Novgorod	nose	R	S	S	S	S	R	S	S	R	S		t127	-	**host**	-	+	-
SA0440REV	*S. aureus*	Lipetsk	skin infection	R	R	R	R	R	R	R	S	R	S		t233	+	-	+	+	+
SA0440ST	*S. aureus*	Nizhny Novgorod	skin wound	R	R	S	R	R	R	S	S	R	S		t008	+	+	+	+	+
SA0445ST	*S. aureus*	Nizhny Novgorod	bones and joints infection	S	S	S	S	S	S	S	S	S	S		t008	+	+	+	+	+
SA0449REV	*S. aureus*	Smolensk	bones and joints infection	R	R	R	R	R	R	R	S	R	S		t008	+	+/-	+	+	+

SA0455ST	*S. aureus*	Nizhny Novgorod	skin wound	R	R	S	R	R	S	S	S	R	S		t331	+	-	+	+	+
SA0462ST	*S. aureus*	Nizhny Novgorod	throat	R	S	S	S	S	R	S	S	R	S		t008	+	+	+	+	+
SA0565REV	*S. aureus*	Kazan	endotracheal tube	S	S	S	S	S	S	S	S	S	S		t9220	+	+	+	+	+
SA0580REV	*S. aureus*	Noyabrsk	blood	S	R	S	S	S	S	S	S	S	S		t267	+	+	+	+	+
SA0592REV	*S. aureus*	Oryol	skin infection	S	S	S	S	S	S	S	S	S	S		t308	+	+	+	+	+
SA0595ST	*S. aureus*	Ryzan	abdominal space infection	R	S	S	S	S	S	S	S	S	S		t308	+	+	+	+	+
SA0617REV	*S. aureus*	Oryol	skin infection	R	R	R	R	R	R	R	S	R	S		t008	+	+	+	+	+
SA0620ST	*S. aureus*	Saint Petersburg	bones and joints infection	R	R	S	R	R	R	S	S	R	S		t435	+	+	+	+	+
SA0621REV	*S. aureus*	Oryol	skin infection	S	R	S	S	S	S	S	S	S	S		t2361	+	+	+	+	+
SA0627REV	*S. aureus*	Smolensk	abdominal space infection	R	R	I	R	R	R	R	S	R	S		t008	+	-	+	+	+
SA0702REV	*S. aureus*	Lipetsk	bones and joints infection	S	S	S	S	S	S	S	S	I	S		t385	+	+	+	+	+
SA0733ST	*S. aureus*	Smolensk	skin wound	R	R	S	S	R	S	S	S	R	S		t308	+	+	+	+	+
SA0736ST	*S. aureus*	Smolensk	abdominal space infection	R	S	S	S	S	S	S	S	S	S		t002	+	+	+	+	+
SA0737ST	*S. aureus*	Smolensk	skin wound	R	S	S	S	S	S	S	S	S	S		t1460	+	+	+	+	+
SA0756ST	*S. aureus*	Smolensk	skin wound	R	S	S	S	R	S	S	S	S	S		t435	+	+	+	+	+
SA0837ST	*S. aureus*	Smolensk	skin wound	S	S	S	S	S	S	S	S	S	S		t008	+	+	+	+	+
SA0855ST	*S. aureus*	Smolensk	skin wound	S	R	S	S	R	S	S	S	R	S		t008	+	-	+	+	+
SA0859ST	*S. aureus*	Smolensk	skin wound	R	R	S	S	S	S	S	S	S	S		t008	+	-	+	+	+
SA0866ST	*S. aureus*	Saint Petersburg	bronchoalveolar lavage	R	S	S	S	S	R	S	S	R	S		t008	+	-	+	+	+
SA2003REV	*S. aureus*	Voronezh	skin infection	S	S	S	S	S	S	S	S	S	S		t308	+	-	+	+	+
SA2014REV	*S. aureus*	Voronezh	skin infection	S	R	S	S	S	S	S	S	S	S		t008	+	+	+	+	+
SA2080REV	*S. aureus*	Rostov-on-Don	skin infection	S	S	S	S	S	S	S	S	S	S		t008	+	-	+	+	+
SA2142REV	*S. aureus*	Chelyabinsk	skin infection	R	R	I	R	R	R	R	S	R	S		N/D	+	+	+	+	+
SA2153REV	*S. aureus*	Smolensk	sputum	S	S	S	S	R	S	S	S	S	S		t002	+	-	+	+	+
SA2242REV	*S. aureus*	Novosibirsk	bones and joints infection	R	R	S	S	S	R	S	S	S	S		t008	+	+	+	+	+
SA2260REV	*S. aureus*	Noyabrsk	blood	S	S	S	S	S	S	S	S	R	S		t521	+	+	+	+	+

SA2263REV	*S. aureus*	Noyabrsk	abdominal space infection	S	R	S	S	S	S	S	S	S	S		t435	+	+	+	+	+
SA2281REV	*S. aureus*	Smolensk	blood	S	S	S	S	S	S	S	S	S	S		t435	+	+	+	+	+
SA2341REV	*S. aureus*	Perm	bones and joints infection	S	S	S	S	S	S	S	S	S	S		t5288	+	+	+	+	+
SA2377REV	*S. aureus*	Perm	bones and joints infection	S	S	S	S	S	S	S	S	S	S		t521	+	+	+	+	+
SA2429REV	*S. aureus*	Lipetsk	skin infection	S	S	S	S	S	S	S	S	S	S		N/D	+	+	+	+	+
SA2442REV	*S. aureus*	Lipetsk	skin infection	R	R	R	R	R	R	R	S	R	S		t008	+	-	+	+	+
SA2464REV	*S. aureus*	Yakutsk	skin infection	S	R	S	S	S	S	S	S	S	S		t002	+	-	+	+	+
SA515	*S. aureus*	Novosibirsk	skin wound	S	S	S	S	S	S	S	S	S	S		t008	**host**	-	+	+	+
SE495	*S. epidermidis*	Moscow	throat	R	R	S	S	S	R	N/D	N/D	S	S	22		+	-	+	+	+
SE0524UT3	*S. epidermidis*	Nizhny Novgorod	urine	S	N/D	S	N/D	N/D	S	S	N/D	N/D	S	57		-	-	-	-	-
SE576	*S. epidermidis*	Moscow	throat	R	R	S	S	S	R	N/D	N/D	S	S	89		-	-	-	-	-
SE580	*S. epidermidis*	Moscow	throat	R	R	S	S	S	R	N/D	N/D	S	S	22		+	-	+	+	+
SE589	*S. epidermidis*	Moscow	fecal masses	R	S	R	R	R	R	N/D	N/D	R	S	269		-	-	-	-	-
SE0003V	*S. epidermidis*	Saint Petersburg	keratoderma infection	R	R	S	N/D	S	I	S	N/D	R	N/D	248		-	-	-	-	-
SE0006V	*S. epidermidis*	Saint Petersburg	conjunctiva	R	R	S	N/D	R	R	R	N/D	S	N/D	59		-	-	-	-	-
SE0019V	*S. epidermidis*	Saint Petersburg	conjunctiva	R	S	R	N/D	R	R	R	N/D	S	N/D	22		+	-	+	+	+
SE0020V	*S. epidermidis*	Saint Petersburg	conjunctiva	S	S	S	N/D	S	S	S	N/D	S	N/D	83		-	-	-	-	-
SE0033V	*S. epidermidis*	Vologda	conjunctiva	S	S	S	N/D	S	S	S	N/D	S	N/D	19		-	-	-	-	-
SE0085V	*S. epidermidis*	Yaroslavl	conjunctiva	R	S	S	N/D	S	S	S	N/D	S	N/D	198		-	-	-	-	-
SE0100V	*S. epidermidis*	Saint Petersburg	conjunctiva	S	R	S	N/D	S	S	S	N/D	S	N/D	6		-	-	-	-	-
SE0103V	*S. epidermidis*	Saint Petersburg	conjunctiva	R	S	R	N/D	R	R	R	N/D	S	N/D	2		-	-	-	-	-
SE0105V	*S. epidermidis*	Saint Petersburg	conjunctiva	R	R	R	N/D	R	R	R	N/D	S	N/D	59		-	-	-	-	-
SE0108V	*S. epidermidis*	Saint Petersburg	conjunctiva	S	S	S	N/D	S	S	S	N/D	S	N/D	19		-	-	-	-	-
SE0109V	*S. epidermidis*	Saint Petersburg	conjunctiva	R	R	S	N/D	S	R	R	N/D	S	N/D	52		-	-	-	-	-

SE0247REV	*S. epidermidis*	Tyumen	blood	R	R	R	R	R	R	R	R	S	S	2		-	-	-	-	-
SE0286UT3	*S. epidermidis*	Moscow	urine	R	N/D	S	N/D	N/D	S	S	N/D	N/D	S	59		-	-	-	-	-
SE0295REV	*S. epidermidis*	Kazan	blood	R	S	S	S	S	R	S	S	S	S	59		-	-	-	-	-
SE0379UT3	*S. epidermidis*	Kazan	urine	R	N/D	S	N/D	N/D	S	S	N/D	N/D	S	59		-	-	-	-	-
SE0382UT3	*S. epidermidis*	Kazan	urine	S	N/D	S	N/D	N/D	S	S	N/D	N/D	S	5		-	-	-	-	-
SE0384UT3	*S. epidermidis*	Kazan	urine	R	N/D	S	N/D	N/D	S	S	N/D	N/D	S	59		+	-	+	+	+
SE0386UT3	*S. epidermidis*	Kazan	urine	S	N/D	S	N/D	N/D	S	S	N/D	N/D	S	86		-	-	-	-	-
SE0419UT3	*S. epidermidis*	Vladivostok	urine	R	N/D	S	N/D	N/D	R	R	N/D	N/D	S	59		-	-	-	-	-
SE0449UT3	*S. epidermidis*	Москва	urine	S	N/D	S	N/D	N/D	S	R	N/D	N/D	S	173		-	-	-	-	-
SE0571REV	*S. epidermidis*	Noyabrsk	blood	R	S	S	S	0,25	S	R	S	S	S	5		-	-	-	-	-
SE178OPN	*S. epidermidis*	Moscow	fecal masses	N/D	N/D	N/D	N/D	N/D	N/D	N/D	N/D	N/D	N/D	59		-	-	-	-	-
SE179OPN	*S. epidermidis*	Moscow	fecal masses	N/D	N/D	N/D	N/D	N/D	N/D	N/D	N/D	N/D	N/D	59		-	-	-	-	-
SE2252REV	*S. epidermidis*	Noyabrsk	blood	S	R	S	S	S	S	S	S	S	S	189		-	-	-	-	-
SE2261REV	*S. epidermidis*	Noyabrsk	blood	R	R	R	R	R	R	R	S	S	S	2		-	-	-	-	-
SE416-2	*S. epidermidis*	Moscow	fecal masses	R	R	S	S	S	R	N/D	N/D	S	S	22		-	-	-	-	-
SE41m	*S. epidermidis*	Moscow	trachea	N/D	N/D	N/D	N/D	N/D	N/D	N/D	N/D	N/D	N/D	2		-	-	-	-	-
SE513	*S. epidermidis*	Moscow	fecal masses	R	R	R	S	S	R	N/D	N/D	S	S	22		-	-	-	-	-
SE517-1	*S. epidermidis*	Moscow	trachea	R	R	S	S	R	R	N/D	N/D	S	S	59		-	-	-	-	-
SE528	*S. epidermidis*	Moscow	throat	R	S	R	S	R	R	N/D	N/D	S	S	2		-	-	-	-	-
SE535 - 1	*S. epidermidis*	Moscow	fecal masses	R	S	S	S	R	R	N/D	N/D	R	S	81		-	-	-	-	-
SE545	*S. epidermidis*	Moscow	fecal masses	R	S	S	S	R	R	N/D	N/D	S	S	59		-	-	-	-	-
SE559	*S. epidermidis*	Moscow	fecal masses	R	R	S	S	R	R	N/D	N/D	R	S	22		+	-	+	+	-
SE564	*S. epidermidis*	Moscow	throat	R	S	S	R	S	R	N/D	N/D	S	S	89		+	-	+	+	+

SE573 - 1	*S. epidermidis*	Moscow	fecal masses	R	R	S	S	R	R	N/D	N/D	R	S	22		-	-	-	-	-
SE573 - 2	*S. epidermidis*	Moscow	fecal masses	R	R	S	S	R	R	N/D	N/D	R	S	81		-	-	-	-	-
SE604	*S. epidermidis*	Moscow	throat	R	R	R	S	R	R	N/D	N/D	S	S	2		-	-	-	-	-
SE646 - 2	*S. epidermidis*	Moscow	throat	R	R	R	S	R	R	N/D	N/D	S	S	59		-	-	-	-	-
SE705	*S. epidermidis*	Moscow	trachea	R	R	S	S	R	R	N/D	N/D	S	S	59		-	-	-	-	-
SE864 - 2	*S. epidermidis*	Moscow	fecal masses	S	N/D	N/D	N/D	N/D	N/D	N/D	N/D	N/D	N/D	59		-	-	-	-	-
SH39	*S. haemolyticus*	Moscow	N/D											17		-	-	-	-	-
SH516	*S. haemolyticus*	Moscow	throat	R	S	R	R	R	R			S	S	17		-	-	-	-	-
SH10V	*S. haemolyticus*	Saint Petersburg	conjunctiva	S	S	S		R	S	S		S		19		-	-	-	-	-
SH18mSt	*S. haemolyticus*	Ulyanovsk	skin and tissues	S		S	S	S			S	S	S	12		-	-	-	-	-
SH21mSt	*S. haemolyticus*	Ulyanovsk	skin and tissues	S		S	S	S			S	S	S	12		-	-	-	-	-
SH240UT	*S. haemolyticus*	Tomsk	urine	S		S			S	S			S	20		-	-	-	-	-
SH29-2St	*S. haemolyticus*	Ulyanovsk	skin and tissues	R		S	S	R			S	R	S	9		-	-	-	-	-
SH38mSt	*S. haemolyticus*	Ulyanovsk	skin and tissues	R		S	S	R			S	R	S	12		-	-	-	-	-
SH582REV	*S. haemolyticus*	Yekaterinburg	blood	R	S	R	S	R	R	R	S	S	S	1		-	-	-	-	-
SH616UT	*S. haemolyticus*	Kazan	urine	S		S			R	S			S	21		-	-	-	-	-
SH622UT	*S. haemolyticus*	Kazan	urine	R		R			R	R			S	7		-	-	-	-	-
SH864-1	*S. haemolyticus*	Moscow	fecal masses											11		-	-	-	-	-
SH421	*S. haemolyticus*	Moscow	throat	R	S	R	S	R	R			S	S	5		-	-	-	-	-
SH568	*S. haemolyticus*	Moscow	throat	R	S	R	S	R	R			S	S	12		-	-	-	-	-

Table S2. The efficiency of plating of bacteriophages vB_SauM-515A1 and vB_SauP-436A1

Strain	Species	EOP of vB_SauM-515A1,%	EOP of vB_SauP-436A1,%
SA0037REV	Staphylococcus aureus	300	67
SA0038REV	Staphylococcus aureus	58	lysis from without
SA0054V	Staphylococcus aureus	1000	2
SA0064BAS	Staphylococcus aureus	267	400
SA0088ST	Staphylococcus aureus	233	167
SA0088V	Staphylococcus aureus	183	2
SA0103BAS	Staphylococcus aureus	250	133
SA0117V	Staphylococcus aureus	lysis from without	lysis from without
SA0120V	Staphylococcus aureus	150	14
SA0156V	Staphylococcus aureus	383	lysis from without
SA0158V	Staphylococcus aureus	283	233
SA0177REV	Staphylococcus aureus	167	117
SA0191REV	Staphylococcus aureus	lysis from without	lysis from without
SA0220V	Staphylococcus aureus	150	200
SA0293REV	Staphylococcus aureus	167	140
SA0346ST	Staphylococcus aureus	150	35
SA0372ST	Staphylococcus aureus	150	230
SA0385REV	Staphylococcus aureus	233	105
SA0395ST	Staphylococcus aureus	183	25
SA0402REV	Staphylococcus aureus	183	83
SA0413REV	Staphylococcus aureus	283	70
SA0422ST	Staphylococcus aureus	233	100
SA436	Staphylococcus aureus	0	100
SA0440ST	Staphylococcus aureus	267	lysis from without
SA0445ST	Staphylococcus aureus	133	lysis from without
SA0449REV	Staphylococcus aureus	233	lysis from without
SA0462ST	Staphylococcus aureus	233	lysis from without
SA0565REV	Staphylococcus aureus	333	67
SA0580REV	Staphylococcus aureus	167	233
SA0592REV	Staphylococcus aureus	250	lysis from without
SA0595ST	Staphylococcus aureus	150	8
SA0617REV	Staphylococcus aureus	200	367
SA0620ST	Staphylococcus aureus	283	lysis from without
SA0621REV	Staphylococcus aureus	300	lysis from without
SA0702REV	Staphylococcus aureus	233	167
SA0733ST	Staphylococcus aureus	100	167
SA0736ST	Staphylococcus aureus	150	57
SA0737ST	Staphylococcus aureus	83	1
SA0756ST	Staphylococcus aureus	117	67
SA0837ST	Staphylococcus aureus	200	lysis from without
SA2014REV	Staphylococcus aureus	200	lysis from without
SA2142REV	Staphylococcus aureus	100	100

SA2242REV	Staphylococcus aureus	300	250
SA2260REV	Staphylococcus aureus	350	50
SA2263REV	Staphylococcus aureus	lysis from without	lysis from without
SA2281REV	Staphylococcus aureus	400	10
SA2341REV	Staphylococcus aureus	100	433
SA2377REV	Staphylococcus aureus	250	167
SA2429REV	Staphylococcus aureus	50	300
SA0515	Staphylococcus aureus	100	0

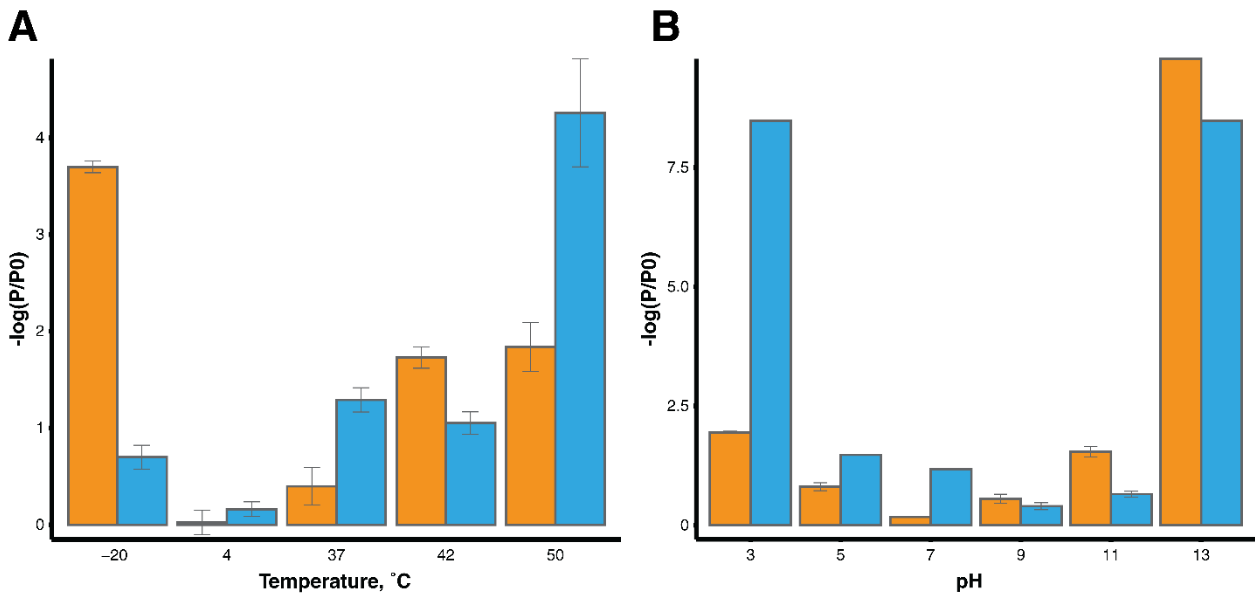

Figure S1. Stability of the vB_SauM-515A1 (orange bars), and vB_SauP-436A1 (blue bars) phages under different temperatures and pH conditions. A: Effect of temperature towards the infection ability of vB_SauM-515A1, and vB_SauP-436A1. A comparison of the titers of bacteriophages at various temperatures was carried out with the titer of the corresponding bacteriophage at 4° C. B: Influence of pH towards the infection ability of vB_SauM-515A1, and vB_SauP-436A1. Phage suspensions were incubated for 24 h at the different pH values and compared with control (pH=8).
